# Terminal Schwann cells at the human neuromuscular junction

**DOI:** 10.1093/braincomms/fcab081

**Published:** 2021-04-15

**Authors:** Abrar Alhindi, Ines Boehm, Rachael O Forsythe, Janice Miller, Richard J E Skipworth, Hamish Simpson, Ross A Jones, Thomas H Gillingwater

**Affiliations:** 1 Edinburgh Medical School: Biomedical Sciences, University of Edinburgh, Edinburgh EH8 9XD, UK; 2 Euan MacDonald Centre for Motor Neurone Disease Research, University of Edinburgh, Edinburgh EH16 4SB, UK; 3 Faculty of Medicine, Department of Anatomy, King Abdulaziz University, Jeddah 22252, Saudi Arabia; 4 Clinical Surgery, Edinburgh Medical School, and Royal Infirmary of Edinburgh, Edinburgh EH16 4SA, UK; 5 Department of Orthopaedic Surgery, University of Edinburgh, Edinburgh EH16 4SB, UK

**Keywords:** neuromuscular junction, terminal Schwann cell, human, mouse, NMJ-morph

## Abstract

Terminal Schwann cells are non-myelinating glial cells localized to the neuromuscular junction. They play an important role in regulating many aspects of neuromuscular junction form and function, in health and during disease. However, almost all previous studies of mammalian terminal Schwann cells have used rodent models. Despite a growing awareness of differences in the cellular and molecular anatomy of rodent and human neuromuscular junctions, it remains unclear as to whether these differences also extend to the terminal Schwann cells. Here, we have adapted immunohistochemical protocols to facilitate visualization and comparative morphometric analyses of terminal Schwann cells at the human and mouse neuromuscular junction. We labelled terminal Schwann cells in the peroneus brevis muscle in six adult mice and five humans with antibodies against S100 protein. All human neuromuscular junctions were associated with at least one terminal Schwann cell, consistent with findings from other species, with an average of ∼1.7 terminal Schwann cells per neuromuscular junction in both humans and mice. In contrast, human terminal Schwann cells were significantly smaller than those of mice (*P* ≤ 0.01), in keeping with differences in overall synaptic size. Human terminal Schwann cell cytoplasm extended significantly beyond the synaptic boundaries of the neuromuscular junction, whereas terminal Schwann cells in mice were largely restricted to the synapse. Moreover, there was a significant difference in the location of terminal Schwann cell nuclei (*P* ≤ 0.01), with human terminal Schwann cells having their nuclear compartment located beyond the perimeter of the synapse more than the mouse. Taken together, these findings demonstrate that terminal Schwann cells at the human neuromuscular junction have notable differences in their morphology and synaptic relationships compared to mice. These fundamental differences need to be considered when translating the findings of both neuromuscular junction biology and pathology from rodents to humans.

## Teaser

Alhindi et al. performed a comparative morphological analysis of terminal Schwann cells at the neuromuscular junction in mice and humans. They report significant species-specific differences that need to be taken into consideration when translating findings between species.

## Graphical Abstract

**Graphical Abstract:**
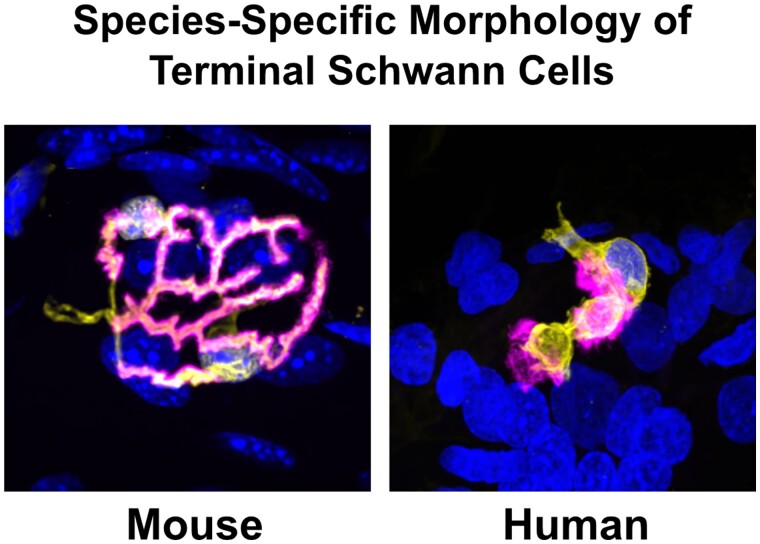

## Introduction

The neuromuscular junction (NMJ) represents the final point of synaptic connection between the peripheral nervous system, in the form of a lower motor neuron, and its target skeletal muscle fibre. As such, the NMJ plays a fundamental role in controlling movement of the body. In addition to this well-described role in the neuromuscular system, the NMJ has been the subject of a renewed research focus, largely due to its contribution to the pathogenesis of a wide range of neuromuscular conditions affecting humans, ranging from motor neuron diseases, such as amyotrophic lateral sclerosis and spinal muscular atrophy, through to autoimmune conditions, such as myasthenia gravis.

Each mammalian NMJ is comprised of a pre-synaptic motor nerve terminal, formed by the lower motor neuron, and the acetylcholine receptor (AChR) enriched motor endplate on the skeletal muscle fibre. In addition to these nerve and muscle components, each NMJ is ‘capped’ by a terminal Schwann cell (tSCs; also known as a peri-synaptic Schwann cell).^1^ These neural crest-derived, non-myelinating glial cells play key roles in regulating the structure and function of the NMJ in health and during disease (see Alvarez-Suarez et al., review).^2^ For example, tSCs are known to mediate developmental processes that direct the formation and maturation of the NMJ.^3^^,^^4^ Similarly, tSCs play important roles in Wallerian degeneration and peripheral nerve regeneration/sprouting after nerve injury.^5^^,^^6^ They also influence age-dependent changes occurring at the NMJ,^7^ and contribute directly to the pathogenesis of conditions such as amyotrophic lateral sclerosis^8^ and Guillain-Barré syndrome.^9^

Despite a growing awareness of the importance of NMJ structure and function in health and disease, the vast majority of our understanding of the NMJ comes from studies on rodent models (mice and rats). Recently, however, comparative studies of the nerve and muscle components of the NMJ in mice and humans have revealed significant species-specific differences at both the cellular and molecular levels.^10^ Human NMJs are significantly smaller, less complex, and more fragmented (‘nummular’) than comparable NMJs in the mouse. Moreover, in stark contrast to previous reports from rodents, human NMJs are remarkably stable across both the normal life-span and in the muscle wasting associated with cancer cachexia.^10^^,^^11^ It remains to be determined whether the cellular and molecular differences between rodent and mouse NMJs result in parallel differences in tSCs. A recent study reported on the number of tSCs per NMJ in vastus lateralis muscles of human,^12^ whilst another reported on tSC changes occurring in ALS patients.^13^ However, studies reporting on comparative morphology of tSCs at the human NMJ are absent from the literature.

To address this important deficiency in our understanding of the cellular composition of the human NMJ, we have developed protocols to allow immunohistochemical labelling, high-resolution imaging and robust morphometric analysis of tSCs at the NMJ in humans. We confirm that tSCs are present at the human NMJ and in similar numbers to those found at the mouse NMJ. In contrast, human tSCs were noted to be significantly smaller than mouse tSCs, with a variable placement of the nucleus in relation to the endplate, and considerably more non-synaptic placement of the tSC cytoplasm than the mouse. Thus, tSCs at the human NMJ have notable differences in their morphology and synaptic relationships compared to tSCs at the mouse NMJ.

## Methods

### Ethics

All human muscle samples were obtained in accordance with the appropriate consent and requisite ethical approvals (NHS Lothian REC: 2002/1/22 and 2002/R/OST/02; NHS Lothian BioResource: SR719, 15/ES/0094 and SR589, 15/SS/0182). All animal work was performed under the appropriate licenses granted by the UK Home Office and within the regulations of the Animals (Scientific Procedures) Act 1986.

### Tissue sampling

Six wild-type mice (C57/BL6, three males and three females, ∼8 weeks old) were euthanised by an overdose of inhaled isoflurane. Within 30 min post-mortem, the peroneus brevis (PB) muscle, one of the muscles of the lateral compartment of hindlimb, innervated by the superficial peroneal nerve, and containing mostly fast-twitch muscle fibres, was dissected out from either side and immediately fixed in 4% paraformaldehyde (PFA) for 30 min. Human samples for tSC analysis were obtained at the same time as our previously published study of human NMJs.^10^ Peroneus brevis (PB) muscle samples were taken from healthy regions of the lower limb in five male patients (mean age = 68.8 years) undergoing surgical amputation for peripheral arterial disease. Within 30 min of the procedure, full-length muscle fibres (2 cm in length) were dissected out from the proximal, healthy end of the discard sample (amputated limb). The health of the sampled tissue was assessed macroscopically (no tissue necrosis, good back bleeding and presence of spontaneous twitching). Microscopically, the health of the NMJs was confirmed by comparison with both previously sampled control tissue^10^ and additional samples of rectus abdominis (RA) muscle, obtained from otherwise healthy, age-matched patients undergoing abdominal surgery (for a full description of the RA sampling method see Boehm et al.).^11^ The RA muscle is one of the anterior abdominal wall muscles, innervated by the lower six thoracic nerves and containing type I and IIa muscle fibres.^14^

### Immunohistochemistry

Samples were immediately fixed in 4% paraformaldehyde for 1–2 h, then washed with 1× phosphate-buffered saline (PBS) before being micro-dissected into small bundles of 10–15 individual fibres. Connective tissue and fat were cleared to reduce potential background staining. Muscle fibres were placed in the following sequence of solutions (made up in 1× PBS unless otherwise specified): glycine for 15 min to reduce tissue auto-fluorescence; 15 min wash in PBS; tetramethyl-rhodamine isothiocyanate-conjugated α-bungarotoxin (TRITC α-BTX; BTIU00012, VWR International Ltd ) 2 µg/ml for 15 min to label AChRs; 4% Triton X-100 for 1.5 h for permeabilization; a blocking solution of 4% bovine serum albumin (BSA) and 2% Triton X-100 for 30 min. Tissue was then incubated with the following primary antibodies overnight at room temperature: ready-to-use rabbit polyclonal anti-S100 IgG (Dako Omnis) which labels S100b strongly, S100A1 weakly, and S100A6 very weakly; mouse anti-S100 antibody (in BSA at 1:100 dilution, ab7852, Abcam); and rabbit monoclonal anti NG2 IgG (neuron-glia protein 2; in BSA at 1:100 dilution, ab255811, Abcam) which labels tSCs; mouse anti-SV2 IgG and mouse anti 2H3 IgG (in BSA at 1:50 dilution, Developmental Studies Hybridoma Bank) to label synaptic vesicles and neurofilaments, respectively; rabbit polyclonal anti myelin basic protein IgG (in BSA at 1:20 dilution, ab2404, Abcam) to label myelin sheath, followed by 1× PBS 4 × 20 min washes. Tissue was next incubated in the following secondary antibodies as needed [AlexaFluor-488-conjugated donkey anti-rabbit IgG (A21206), AlexaFluor-680-conjugated donkey anti-rabbit IgG antibody (A10043), AlexaFluor-488-conjugated donkey anti-mouse IgG antibody (a21202), all in 1× PBS at 1:400 dilution, Life Technologies] overnight at 4°C or 5 h at room temperature, followed by 4 × 20 min washes with 1× PBS; with final DAPI staining for 15 min (1:1000) followed by 1× PBS 3 × 10 min washes. Muscle fibres were mounted on a glass slide in Mowiol. Samples were protected against photobleaching by dark storage wherever possible.

### Confocal imaging

A Nikon A1R FLIM confocal laser scanning microscope with 60×/1.4 oil immersion objective was used to capture 16-bit, 512 × 512 pixel frame size, Z—stack images with 0.5 µm interval, at 2× zoom; red channel—561 nm excitation; green channel—488 nm excitation; blue channel—405 nm excitation. A minimum of 17 NMJs (*en face* or <10° oblique) were acquired per muscle in each species.

### Morphological analyses

Fiji software was used for quantitative analysis of confocal micrographs. All analyses were performed on maximum intensity projections. In total, 8 different morphological variables were quantified (Table 1). The number of tSCs per NMJ was counted manually, whilst the remainder of the variables were analysed using our established NMJ-morph/aNMJ-morph methodology,^15^^,^^16^ with a few modifications to make it suitable for tSC analysis instead of motor nerve terminals. As the NMJ region also contains an abundance of myonuclei, tSCs were positively identified and only included in analyses if a nucleus (DAPI staining) was located within a cytoplasmic halo (S100 labelling) and was positioned over or near the endplate (TRITC α-BTX labelling). Other tSC variables included size-related parameters (area and perimeter), the relationship of tSC processes to the post-synaptic component (coverage and extension), and the placement of tSC nuclei in relation to AChRs (synaptic and non-synaptic). The measurement of individual variables and their rationale for inclusion is discussed in detail below (see Results). In addition, post-synaptic measurements were also recorded, including AChR area and perimeter, number of AChR clusters and endplate fragmentation.^15^^,^^16^

**Table 1:** Summary of morphometric data (human tSC versus mouse tSC)

	Mouse	Human
	*N* = 6; *n* = 126 NMJ	*N* = 5; *n* = 151 NMJ
	214 tSCs	247 tSCs
Number of tSCs per NMJ	1.74 ± 0.06	1.73 ± 0.07
Total tSC perimeter (μm)	280.80 ± 8.92^**^	178.30 ± 6.14
Total tSC area (μm^2^)	258.51 ± 8.48^**^	199.15 ± 7.11
Synaptic area of tSCs (μm^2^)	184.33 ± 6.07^****^	89.27 ± 3.86
Coverage (%)	68.89 ± 0.71^*^	54.14 ± 1.06
Non-synaptic area of tSCs (μm^2^)	74.17 ± 3.47^****^	109.88 ± 4.79
Extension (%)	28.23 ± 0.86^**^	54.55 ± 1.27
Unoccupied area of AChR (μm^2^)	82.54 ± 2.8	65.65 ± 2.84

In total, 8 separate morphological variables were measured. Values are mean ± SEM (standard error of mean). See Fig. 2 for explanation of derived terms (synaptic area, non-synaptic area, coverage, extension). Unpaired *t*-test for parametric variables; Mann–Whitney test for non-parametric variables.

* *P* ≤ 0.05,

** *P* ≤ 0.01,

**** *P* ≤ 0.0001. AChR, acetylcholine receptors; NMJ, neuromuscular junction; tSCs, terminal Schwann cells.

### Statistical analyses

Statistical analyses for species comparisons were performed using an unpaired *t*-test for parametric data, or Mann–Whitney test for non-parametric data. Correlation analyses for post-synaptic and tSC variables were performed using Pearson’s or Spearman’s correlation coefficients. GraphPad Prism Software (Version 8) was used for all statistical analyses. Data are reported as mean ± SEM. Individual tests are referenced in their corresponding figure legends. *P*-values: * ≤ 0.05, ** ≤ 0.01, *** ≤ 0.001, **** ≤ 0.0001.

### Data availability

The data that support the findings of this study are available on request from the corresponding author. The data are not publicly available due to privacy or ethical restrictions.

## Results

To enable high-resolution comparative morphometric analyses of human and mouse terminal Schwann cells (tSCs), we modified our existing tissue sampling and immunohistochemical staining approaches to examine human NMJs in samples of healthy peroneus brevis (PB) muscle obtained from patients undergoing lower limb amputation surgery.^10^^,^^11^^,^^17^ Importantly, as previously demonstrated, samples were obtained from otherwise healthy regions of the limb, devoid of any pathological changes resulting from the underlying conditions that necessitated surgical amputation.^10^^,^^11^

**Figure 1:**
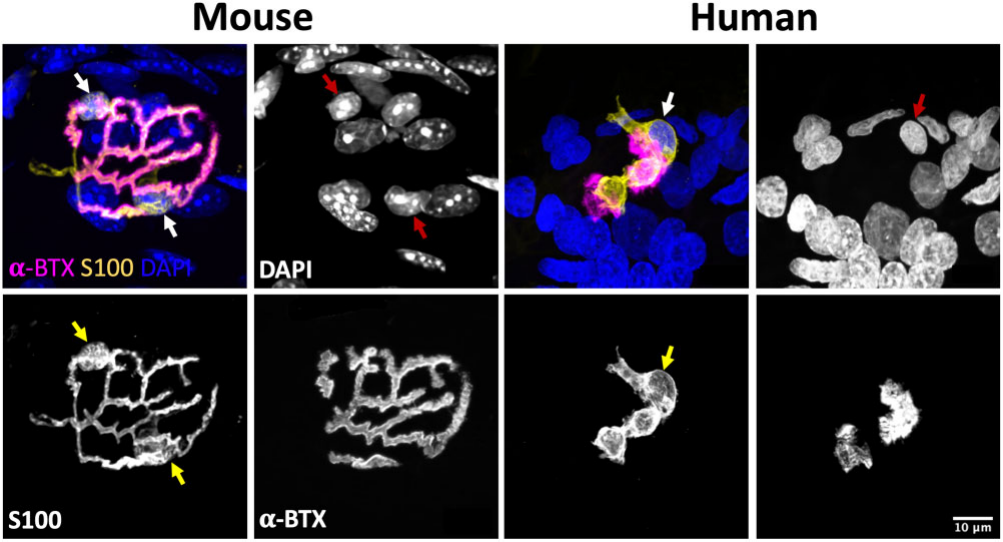
**Terminal Schwann cells at the mouse and human neuromuscular junction**. Representative confocal micrographs of mouse and human NMJs. Merged images show tSCs (yellow), AChRs (magenta) and nuclei (blue). tSC nuclei (red arrows) are identified by the surrounding halo of S100 cytoplasm (yellow arrows). Note how mouse tSCs closely mirror their corresponding AChR profiles; human tSCs show much less congruence, including non-synaptic cytoplasm that does not directly overlie the motor endplate. Terminal Schwann cells (tSCs) labelled with S100 (yellow); acetylcholine receptors (AChRs) labelled with α-BTX (magenta); nuclear staining with DAPI (blue). Scale bar = 10 µm across all the images.

Once protocols for S100 immunohistochemistry had been optimized to allow parallel, high-resolution confocal imaging of both human and mouse PB samples, we began by undertaking a qualitative analysis of tSCs in humans and mice (Fig. 1). Initial observations revealed that tSCs were found at all mouse and human NMJs examined. The appearance of tSCs at the mouse NMJ were very similar to those previously described in the literature: tSC somata were clearly identifiable above the motor endplate, with the tSC cytoplasm closely mirroring the location and distribution of the motor nerve terminals.^18–20^ However, consistent and notable differences between human and mouse tSCs were immediately apparent (Fig. 1). Cytoplasmic processes of mouse tSCs closely mirrored the patterning of their underlying AChRs (and therefore the patterning of the motor nerve terminals), being almost perfectly aligned with their neighbouring AChRs and rarely extending beyond their edges. In contrast, cytoplasmic processes of human tSCs rarely mirrored the pattern of their neighbouring AChRs, and they seldom covered the whole synaptic area.

**Figure 2:**
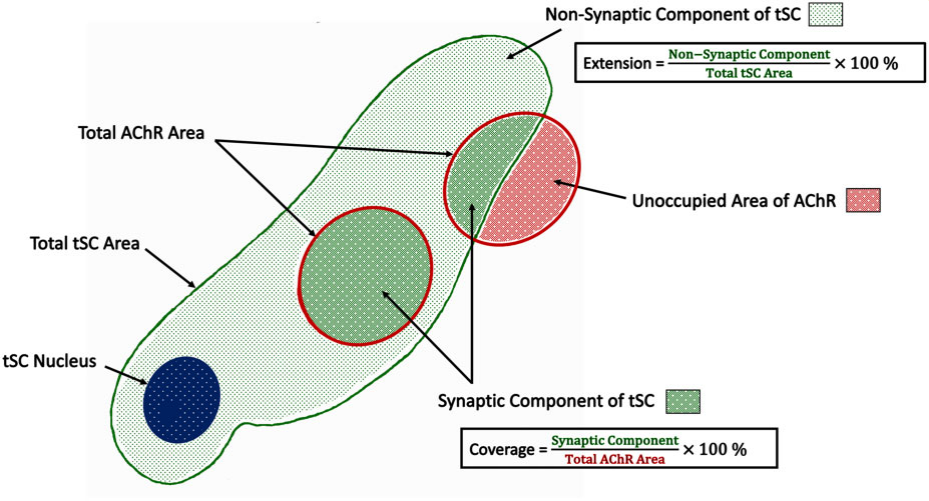
**Morphometric analysis of terminal Schwann cells**. Schematic diagram. In addition to basic measurements of area and perimeter, several ‘markers of congruence’ were also defined. The total area of the terminal Schwann cell was sub-divided into a ‘synaptic component’ (directly overlying the AChRs) and a ‘non-synaptic component’ (extending beyond the AChRs). These measurements were then used to derive the percentage ‘extension’ of the terminal Schwann cells (beyond the AChRs) and the percentage ‘coverage’ of the AChRs (by the terminal Schwann cells). See also Table 1.

In order to build upon these initial qualitative observations, we next performed a quantitative analysis of tSC morphology at mouse and human NMJs. In total, we analysed 8 individual morphological variables across 126 mouse NMJs and 151 human NMJs, using a modification of our established NMJ-morph/aNMJ-morph workflow.^15^^,^^16^ In addition to basic variables such as tSC area and perimeter, we defined several ‘markers of congruence’ to quantify the precise spatial relationship between tSCs and their underlying synaptic AChRs (Fig. 2; Table 1).

**Figure 3:**
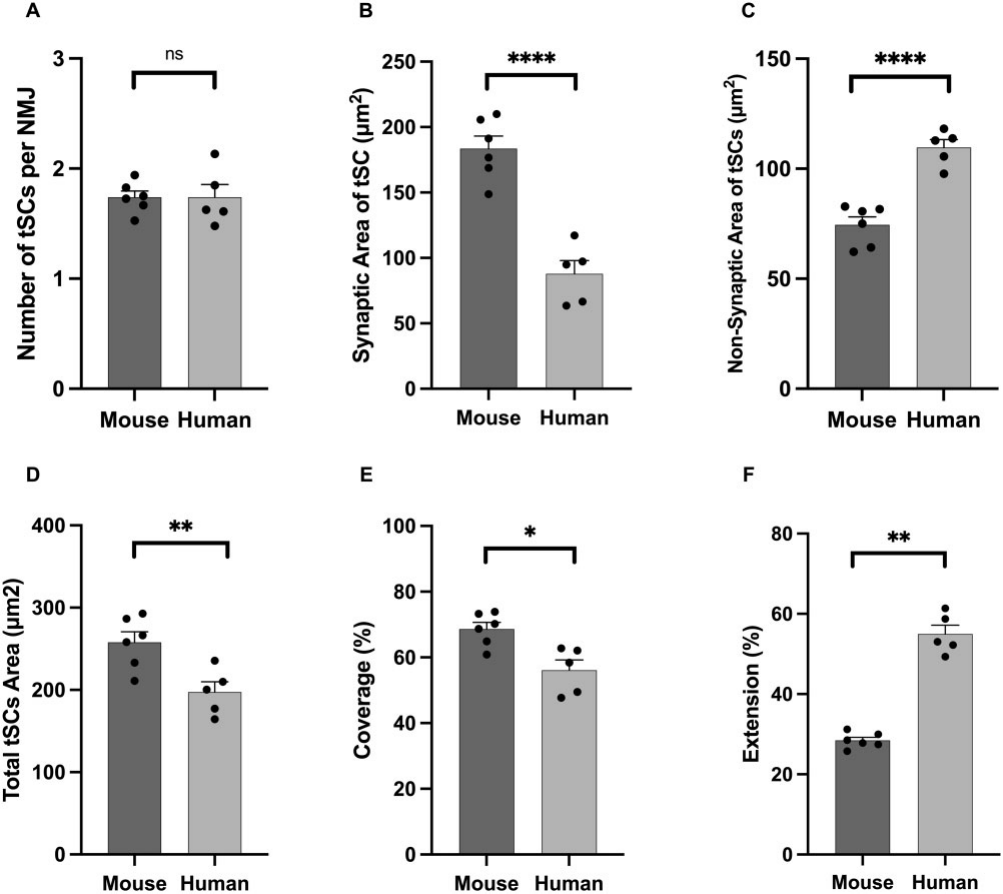
**Species-specific differences in terminal Schwann cell morphology**. Comparative analysis revealed characteristic differences in overall tSC morphology. Although the number of tSCs (per NMJ) was similar in both species (**A**), human tSCs were significantly smaller than those of mice (**D**). Characteristic differences were also noted in the spatial relationship between tSC and motor endplate (‘markers of congruence’ – **B, C, E, F**), with human tSCs having less ‘coverage’ of the AChRs (∼50% human cf. ∼70% mouse; panel E) but greater ‘extension’ beyond them (∼60% human cf. ∼30% mouse; panel F). Bar charts are mean ± SEM; each data point represents an individual muscle (human PB; *N* = 5, mouse PB; *N* = 6; a minimum of 17 NMJs per muscle; in total *n* = 126 mouse NMJs; *n* = 151 human NMJs). Unpaired *t*-test for parametric variables; Mann–Whitney test for non-parametric variables. *P ≤ 0.05, **P ≤ 0.01, ****P ≤ 0.0001.

As with our qualitative observations, quantitative analysis confirmed that every NMJ was associated with at least one tSC in both mice and humans. Although human NMJs are significantly smaller than mouse NMJs,^10^ there was no significant difference in the average number of tSCs per NMJ between humans and mice (∼1.7 tSCs/NMJ; Fig. 3A;Table 1). Thus, the total number of tSCs recruited to an NMJ is not influenced by the absolute size of the associated synaptic ‘footprint’. In contrast, markedly distinct differences were noted in all other morphological variables, with mouse tSCs having a significantly larger cellular area/perimeter than human tSCs (Fig. 3D;Table 1).

A direct comparison of the spatial relationship between tSCs and their underlying AChRs revealed further species-specific differences. More than 50% of the total area of human tSCs extended beyond the boundaries of the corresponding AChRs, compared to <30% in mice (Fig. 3F;Table 1). Thus, even though human tSCs were significantly smaller than mouse tSCs, a larger proportion of their cytoplasm was not to be found directly overlying the area of synaptic contact between nerve and muscle. This was reflected in both increased non-synaptic area and decreased synaptic area, of human tSCs compared to mice (Fig. 3B and C; Table 1) with a significant reduction in the percentage coverage of AChRs by human tSCs (Fig. 3E;Table 1). Interestingly, however, portions of the AChR region were notably devoid of any overlying tSC cytoplasm in both human and mouse NMJs (Fig. 1; Table 1).

**Figure 4:**
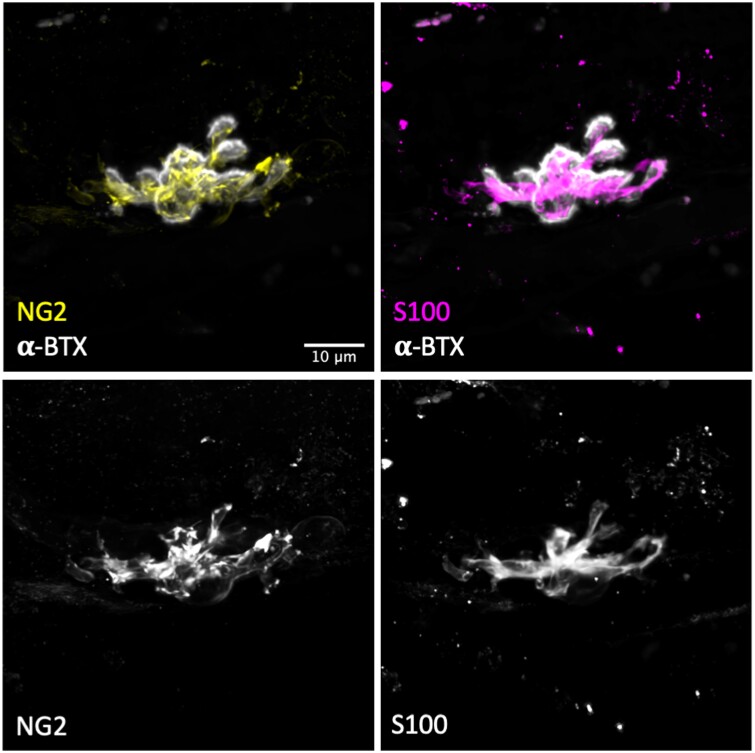
**S100 is a reliable marker for human tSCs**. Representative confocal micrographs of human tSCs from the PB muscle with double immunolabelling to show co-localization of anti NG2 (yellow) and anti-S100 (magenta), confirming that S100 is an accurate marker for human tSCs. Both markers (S100-magenta) and (NG2-yellow) showed similar staining patterns and revealed similar tSC morphology. S100 staining was found to be more intense and more evenly distributed within the cell (particularly within the tSC cytoplasm). Acetylcholine receptors (AChRs) labelled with α-BTX (grey). Scale bar = 10 µm across all images.

Next, in order to confirm that the labelling of human tSCs using S100 was revealing the true morphology of the cells, we utilized a second tSC marker: NG2. NG2 has previously been shown to be co-expressed with S100 in mouse tSCs.^21^ Similar to previous reports from rodents, we found that S100 and NG2 were co-expressed at human tSCs (Fig. 4). Although tSC morphology was virtually indistinguishable between the two markers (S100 and NG2), the staining intensity was greater using antibodies against S100, making it more reliable for quantitative analyses.

**Figure 5:**
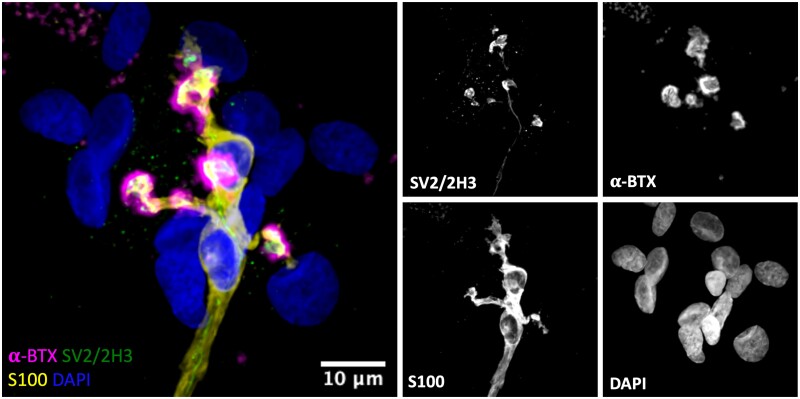
**Close congruence between motor nerve terminal and tSCs at the human NMJ**. Representative confocal micrograph of a human NMJ from the PB muscle. Triple labelling of the basic cellular components of the NMJ; skeletal muscle fibre (AChRs; magenta), motor nerve terminals (green) and tSCs (yellow). The micrograph demonstrates a typical ‘healthy’ NMJ—the endplate is fully innervated, and there is close congruence between the nerve terminals and tSCs. The merged image shows terminal Schwann cells (tSCs) labelled with antibodies against S100 (yellow), nerve terminals labelled with antibodies against 2H3 and SV2 (green), acetylcholine receptors (AChRs) labelled with α-BTX (magenta), and nuclear staining with DAPI (blue). Scale bar = 10 µm.

We next evaluated the relationship between tSCs and their respective nerve terminals and myelin sheaths at the human NMJ. As expected, we found that all human endplates were fully innervated by an incoming lower motor neuron, with the morphology of tSCs closely matching their respective nerve terminals (Fig. 5). Labelling of Schwann cell-generated myelin using antibodies against myelin basic protein confirmed that human tSCs were non-myelinating, with the intact myelin sheath surrounding the preterminal axon terminating well before the axon branched into the motor nerve terminals (Supplementary Fig. 1).

**Figure 6:**
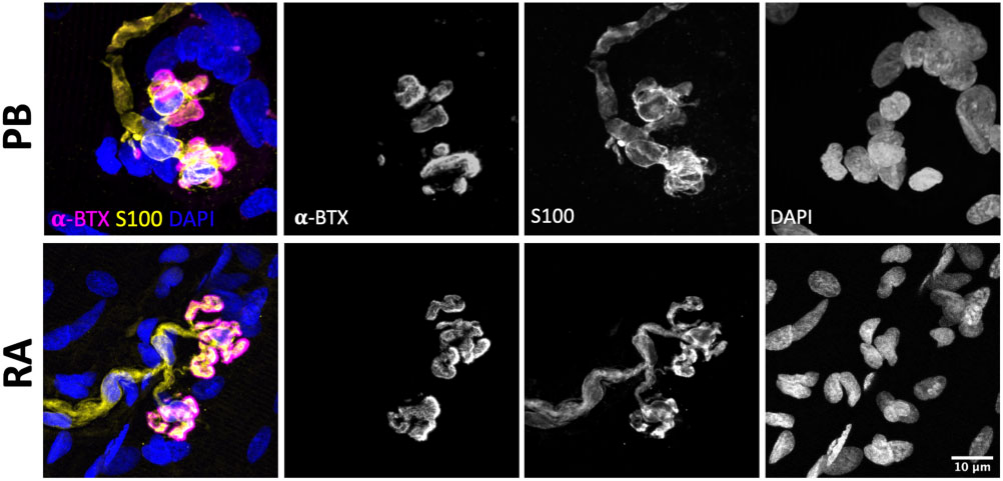
**Consistent tSC morphology across different human muscles**. Representative confocal micrographs of human NMJs obtained from PB (peroneus brevis; top panels) and RA (rectus abdominus; bottom panels). PB muscle samples were harvested from patients undergoing lower limb amputation; RA muscle biopsies were obtained from patients undergoing abdominal surgery. tSCs had a very similar appearance in both muscles, suggesting that the morphology reported in PB samples was not related to body region, patient pathology, and/or sampling technique. Merged images show terminal Schwann cells (tSCs) labelled with S100 (yellow), acetylcholine receptors (AChRs) labelled with α-BTX (magenta), and nuclear staining with DAPI (blue). Scale bar = 10 µm across all images.

In order to confirm that the observed morphology of human tSCs reported in the PB muscle was reflective of ‘normal’ and ‘healthy’ tSCs, and was not modified as a consequence of the muscle identify/body region, underlying pathology in the patients, and/or sampling techniques utilized during lower limb amputation, we also examined tSC morphology in rectus abdominis (RA) muscle samples that were collected from two otherwise healthy, age-matched patients undergoing abdominal surgery. A total of 52 NMJs were inspected from these RA specimens, with no overt differences to note between their tSC morphology and that previously reported from PB samples (Fig. 6). It is also important to note that we did not observe any obvious signs of pathological ageing or denervation in human NMJs from our PB muscle samples, where all NMJs examined revealed a typical ‘healthy’ NMJ morphology, with no evidence of nerve terminal sprouting or retraction, and with no tSC sprouting to adjacent endplates.^20^^,^^22–24^

**Figure 7:**
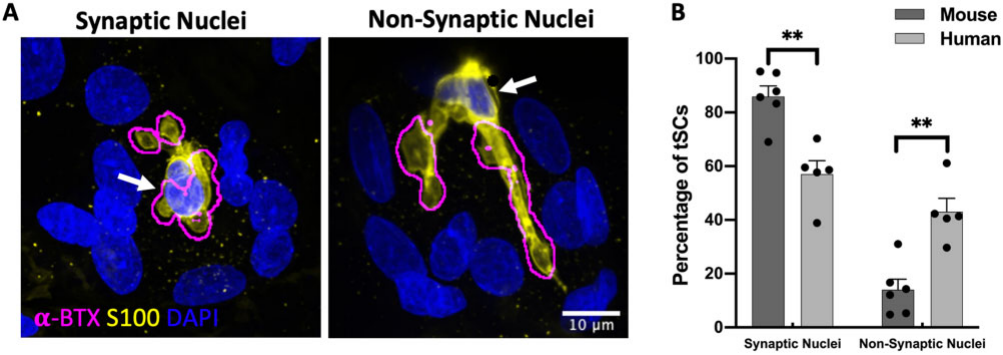
**Location and placement of nuclei in human terminal Schwann cells**. (**A**) Representative micrographs of human NMJs illustrating ‘synaptic’ and ‘non-synaptic’ placement of tSC nuclei (yellow: tSC/S100, blue: nuclei/DAPI). The ‘Find Edges’ function in ImageJ/FIJI was applied to the magenta channel (AChRs/alpha-BTX) to define the boundaries of the AChRs (methods acquired from Carrasco et al.^23^). The location of tSC nuclei is indicated by the white arrows. (**B**) Although the vast majority of mouse tSCs had a ‘synaptic’ placement of their nuclei (∼80%), human tSCs revealed a more even balance of ‘synaptic’ (∼60%) and ‘non-synaptic’ (∼40%) nuclei. Note also the ‘annular’ arrangement of myonuclei around the human NMJ. Bar charts are mean ± SEM; each data point represents an individual muscle; mouse: *N* = 6; *n* = 214 tSCs; human: *N* = 5; *n* = 247 tSCs. Mann–Whitney test for non-parametric variables. **P ≤ 0.01. Scale bar = 10 µm in both images.

Our qualitative analyses of human and mouse NMJs suggested that there were differences in the location of tSC nuclei (and thus the position of the cell body) in humans and mice. Therefore, we quantified the number of tSCs with either a ‘synaptic’ nucleus (overlying the AChRs/endplate) or a ‘non-synaptic’ nucleus (not overlying the AChRs/endplate) (Fig. 7), and we found that the number of tSCs with ‘synaptic’ nuclei was significantly higher in mouse compared to the human (Fig. 7B). Whilst the majority of mouse tSCs (∼80%) had ‘synaptic’ nuclei, human tSCs tended to demonstrate a more even balance between ‘synaptic’ (∼60%) and ‘non-synaptic’ (∼40%) nuclei. Thus, the spatial relationship between the cell soma of a tSC and its associated NMJ is fundamentally different at the human NMJ compared to the mouse NMJ.

**Figure 8:**
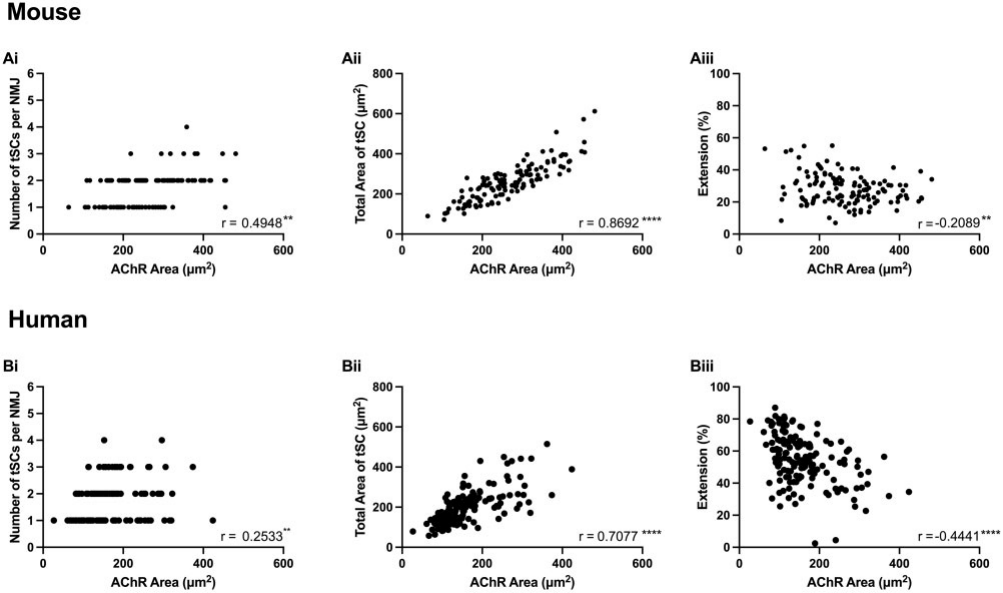
**Relationship between NMJ size and terminal Schwann cell morphology**. Correlation analyses from both mouse (upper panels) and human (lower panels) NMJs. A significant, albeit modest, correlation was observed between NMJ size (AChR area) and tSC number in both mice (**Ai**) and humans (**Bi**). However, a stronger correlation was present between NMJ size and tSC area in both species (**Aii** and **Bii**). Also, an increase in AChR area was associated with a reduction in tSCs cytoplasmic extension (**Aiii** and **Biii**). Each data point represents a single NMJ and its tSCs (*n* = 126 mouse NMJs; *n* = 151 human NMJs). Pearson and Spearman correlation coefficients (*r*) for parametric and non-parametric variables, respectively. **P ≤ 0.01; ****P ≤ 0.0001.

Finally, in order to investigate factors potentially regulating the number and size of tSCs at the mammalian NMJ, we performed a series of correlation analyses comparing NMJ and tSC variables in humans and mice (Fig. 8). We found a significant (albeit modest) correlation between the size of an NMJ (AChR area) and the number of accompanying tSCs in both mice and humans (Fig. 8Ai and Bi). However, the strength of correlation was lower in humans than in mice (mouse *r* = 0.49 versus human *r* = 0.25), suggesting that the relative influence of NMJ size on the number of tSCs present was weaker in humans than in mice. This is in keeping with our finding of similar numbers of tSCs at human and mouse NMJs, even when mouse NMJs were significantly larger (Fig. 3A). Moreover, previous studies in rodents have suggested a strong correlation between NMJ size and the number of tSCs present.^18^^,^^25^^,^^26^ However, in both species, total tSC area was strongly correlated with the area of AChRs at the NMJ (Fig. 8Aii and Bii), with a significant reduction in cytoplasmic extension (and thus greater congruence between tSC and AChRs at larger NMJs) similarly observed (Fig. 8Aiii and Biii). Taken together, these observations suggest that the mechanisms regulating the *morphology* of tSCs at the NMJ are likely to be similar between the two species, whereas those that regulate the relative *number* of tSCs at each NMJ show more species-specific differences.

## Discussion

TSCs have vital roles to play at the NMJ during development, maturation and after nerve injury or disease.^27^ In the current study, we have extended our understanding of species-specific differences at the mammalian NMJ^10^^,^^17^ to show that significant differences are also present in tSC morphology between humans and mice. Despite having similar numbers of tSCs per NMJ, human tSCs were significantly smaller and characterized by a larger non-synaptic area, less AChR coverage, and the presence of both synaptic and non-synaptic placement of nuclei.

Although NMJs in mice are significantly larger than those in humans,^10^ NMJs of both species were associated with a similar number of tSCs. This surprising finding suggests that a single tSC in mice has the capacity to support and ‘cap’ a larger synaptic area than equivalent tSCs in humans. The reasons for this remain unclear. However, it is not the case that the absolute number of tSCs at the mammalian NMJ are fixed and consistent between all species, as higher numbers have been reported at adult rat NMJs^18^^,^^19^ and the number of tSCs can be significantly altered by the effects of pathology (e.g. amyotrophic lateral sclerosis, spinal muscular atrophy and Duchenne muscular dystrophy).^23^^,^^25^^,^^26^

It has been shown that tSCs play important roles in regulating transmission and synaptic stabilization at the NMJ,^27^ which suggests that an increase in NMJ size is likely to require a similar/matched increase in the number or size of tSCs in order to adequately cover and support the synapse. Our data support this notion, as in both species increased AChR area correlated with a robust increase in the size of tSCs. This finding supports previous studies demonstrating a dynamic relationship between NMJ size and tSCs in rodents. For example, a mouse study found that growth of the motor endplate was accompanied by an increase in tSC area, rather than number.^20^ However, a significant correlation was found between tSC number and endplate area in rats, both during development and in adulthood.^18^ Moreover, this relationship was found to hold when endplate size was manipulated by altering testosterone levels^18^ or when it was affected by disease pathology.^25^^,^^26^

Finally, our finding that human tSCs tend to have more non-synaptic placement of their nuclei, and less coverage of neighbouring AChRs, is of potential importance for interpreting pathological changes at the NMJ, as these two morphological features have previously been associated with pathological changes at the NMJ in rodent models. For example, in ALS mice, a large proportion of tSCs were found to have non-synaptic nuclei compared to control animals.^23^ In addition, some endplates were partially covered while others showed a complete absence of S100 labelling over the endplate.^23^ Similarly, following denervation experiments in mice, up to 28% of AChRs were found to be completely devoid of any overlying tSC processes.^28^ Thus, the features of ‘pathological’ tSCs identified and characterized in rodent models are actually ‘normal’ features of tSCs at the human NMJ. These observations highlight the need to fully understand the species-specific features of tSC structure and function, and take this into consideration when translating scientific and pre-clinical research findings across different mammalian species.

Many studies have highlighted fundamental differences in the molecular composition and functional capacity between mouse and human Schwann cells (SCs). Some of these differences show that human SCs have normally low expression of glia fibrillary acidic protein and other proteins that are upregulated in rodents during nerve regeneration, such as adhesion molecules and nerve growth factor receptor. Another distinguishable feature was the early appearance of senescence-like morphology in human SC culture relative to rodents. They were also harder to maintain in neuron-SC co-culture, showed lower proliferation and differentiation rate, and failed to extend processes or form myelin sheaths.^29–31^ Overall, these studies suggest that human SCs are unique and that the findings from experimental animals do not necessarily reflect the nature of human SCs. Our work extends the understanding of such species-specific differences in SCs to reveal similar fundamental differences of *in situ* tSC morphology between human and mouse NMJs.

Taken together with our recently published comparative study of mammalian NMJ morphology,^17^ it is clear that considerable heterogeneity of NMJ morphology exists both within and between mammals, including humans. Future studies combining a range of morphological and physiological techniques—such as high-resolution imaging, muscle fibre typing, and electrophysiology—will now be required to ascertain the key determinants of structure–function relationships at the NMJ in both health and disease.

## Supplementary material

Supplementary material is available at *Brain Communications* online.

## Funding

This study was supported by a Prize PhD Studentship from the Anatomical Society (to I.B. and T.H.G.), PhD funding from King Abdulaziz University through the Saudi Cultural Bureau, London (to A.A.), and small project grant funding from the Royal College of Surgeons of Edinburgh (to J.M., R.S., and R.A.J.). R.S. is supported by an NHS Research Scotland (NRS) Clinician post.

## Competing interests

The authors have no conflicts of interest to declare.

## Supplementary Material

fcab081_Supplementary_Data

## Abbreviations

α-BTX: alpha-bungarotoxin
AChR: acetylcholine receptors
BSA: bovine serum albumin
NMJ: neuromuscular junction
PB: peroneus brevis
PBS: phosphate-buffered saline
RA: rectus abdominis
SCs: Schwann cells
tSCs: terminal Schwann cells
TRITC: tetramethyl-rhodamine isothiocyanate
