# Supplementary material for: Terminal Schwann cells at the human neuromuscular junction
Source: Brain Commun. 2021 Apr 15;3(2):fcab081. doi: 10.1093/braincomms/fcab081 (PMC8093923; doi:10.1093/braincomms/fcab081)
Supplement: fcab081_Supplementary_Data [file fcab081_supplementary_data.pdf]

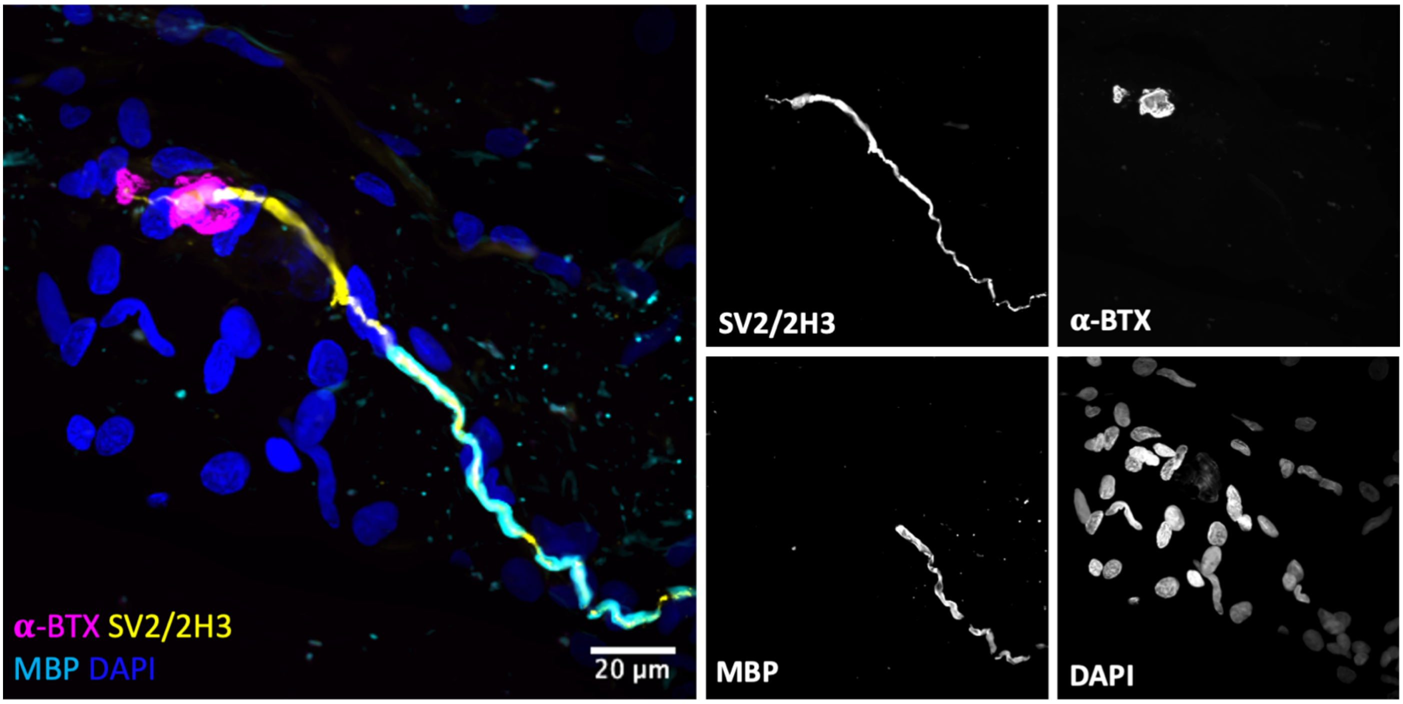

Supplementary Figure 1: Human tSCs are non-myelinating.

Representative confocal micrograph of a human NMJ obtained from PB muscle labelled with MBP (a myelin marker). A myelin sheath can be seen surrounding the preterminal axon, but this terminates some distance (~20-40 μm) from the endplate, but there is no evidence of MBP labelling in relation to the NMJ itself, suggesting that human tSC are non-myelinating. Merged image shows the myelin sheath labelled with antibodies against MBP (cyan), nerve terminals labelled with antibodies against SV2/2H3 (yellow), acetylcholine receptors (AChRs) labelled with α-BTX (magenta) and nuclear staining with DAPI (blue). Scale bar = 20 μm.
